# A full genome assembly reveals drought stress effects on gene expression and metabolite profiles in blackcurrant (*Ribes nigrum* L.)

**DOI:** 10.1093/hr/uhae313

**Published:** 2024-11-11

**Authors:** Freya Maria Rosemarie Ziegler, Vivien Rosenthal, Jose G Vallarino, Franziska Genzel, Sarah Spettmann, Łukasz Seliga, Sylwia Keller-Przybyłkowicz, Lucas Munnes, Anita Sønsteby, Sonia Osorio, Björn Usadel

**Affiliations:** CEPLAS, Institute of Bio- and Geosciences (IBG-4: Bioinformatics) & Bioeconomy Science Center (BioSC), Forschungszentrum Jülich, Wilhelm-Johnen-Straße, D-52425 Jülich, Germany; Faculty of Mathematics and Natural Sciences, CEPLAS, Institute for Biological Data Science, Universitätsstr. 1, D-40225 Düsseldorf, Germany; Cluster of Excellence on Plant Sciences (CEPLAS), Heinrich Heine University Düsseldorf, Universitätsstr. 1, D-40225 Düsseldorf, Germany; Faculty of Mathematics and Natural Sciences, CEPLAS, Institute for Biological Data Science, Universitätsstr. 1, D-40225 Düsseldorf, Germany; Cluster of Excellence on Plant Sciences (CEPLAS), Heinrich Heine University Düsseldorf, Universitätsstr. 1, D-40225 Düsseldorf, Germany; Departamento de Biología Molecular y Bioquímica, Campus de Teatinos, Instituto de Hortofruticultura Subtropical y Mediterránea ‘La Mayora’, Universidad de Málaga-Consejo Superior de Investigaciones Científicas, 29010 Málaga, Spain; CEPLAS, Institute of Bio- and Geosciences (IBG-4: Bioinformatics) & Bioeconomy Science Center (BioSC), Forschungszentrum Jülich, Wilhelm-Johnen-Straße, D-52425 Jülich, Germany; CEPLAS, Institute of Bio- and Geosciences (IBG-4: Bioinformatics) & Bioeconomy Science Center (BioSC), Forschungszentrum Jülich, Wilhelm-Johnen-Straße, D-52425 Jülich, Germany; Department of Plant Crop breeding, INHORT, National Institute of Horticultural Research, Konstytucji 3 Maja 1/3, 96-100 Skierniewice, Poland; Department of Plant Crop breeding, INHORT, National Institute of Horticultural Research, Konstytucji 3 Maja 1/3, 96-100 Skierniewice, Poland; Faculty of Mathematics and Natural Sciences, CEPLAS, Institute for Biological Data Science, Universitätsstr. 1, D-40225 Düsseldorf, Germany; NIBIO, Norwegian Institute of Bioeconomy Research, Division of Food Production and Society Horticulture, Pb. 115, NO-1431 Ås, Norway; Departamento de Biología Molecular y Bioquímica, Campus de Teatinos, Instituto de Hortofruticultura Subtropical y Mediterránea ‘La Mayora’, Universidad de Málaga-Consejo Superior de Investigaciones Científicas, 29010 Málaga, Spain; CEPLAS, Institute of Bio- and Geosciences (IBG-4: Bioinformatics) & Bioeconomy Science Center (BioSC), Forschungszentrum Jülich, Wilhelm-Johnen-Straße, D-52425 Jülich, Germany; Faculty of Mathematics and Natural Sciences, CEPLAS, Institute for Biological Data Science, Universitätsstr. 1, D-40225 Düsseldorf, Germany; Cluster of Excellence on Plant Sciences (CEPLAS), Heinrich Heine University Düsseldorf, Universitätsstr. 1, D-40225 Düsseldorf, Germany

## Abstract

Blackcurrant (*Ribes nigrum* L., family Grossulariaceae) is a perennial shrub that is widely cultivated for its edible berries. These are rich in antioxidants, vitamin C, and anthocyanins, making them a valuable ingredient in the food and beverage industry. However, prolonged periods of drought during the fruiting season lead to drought stress, which has serious ecological and agricultural implications, inhibiting blackcurrant growth and reducing yields. To facilitate the analysis of underlying molecular processes, we present the first high-quality chromosome-scale and partially haplotype-resolved assembly of the blackcurrant genome (cv. Rosenthals Langtraubige), also the first in the family Grossulariaceae. We used this genomic reference to analyze the transcriptomic response of blackcurrant leaves and roots to drought stress, revealing differentially expressed genes with diverse functions, including those encoding the transcription factors *bZIP*, *bHLH*, *MYB*, and *WRKY*, and tyrosine kinase-like kinases such as *PERK* and *DUF26*. Gene expression was correlated with the abundance of primary metabolites, revealing 14 with significant differences between stressed leaves and controls indicating a metabolic response to drought stress. Amino acids such as proline were more abundant under stress conditions, whereas organic acids were depleted. The genomic and transcriptomic data from this study can be used to develop more robust blackcurrant cultivars that thrive under drought stress conditions.

## Introduction

Blackcurrant (*Ribes nigrum* L.) is a winter-hardy shrub (family Grossulariaceae) cultivated in temperate regions mainly for its edible berries. These are associated with several health benefits reflecting the high levels of vitamin C, anthocyanins, antioxidants, and flavonoids. However, many blackcurrant cultivars produce less floral biomass and abort a greater proportion of flowers in response to drought stress, reducing the commercial berry yield [3, 4].

Drought stress affects multiple biochemical and physiological processes at the cellular and whole-plant levels, including growth, membrane integrity, pigment synthesis, osmotic adjustment, water relations, and photosynthetic activity [4]. It also promotes senescence, which is necessary for plant survival. Leaf senescence requires the combined activity of phytohormones and transcription factors such as those of the *NAC*, *AP2/ERF*, and *WRKY* families, which are modulated by ethylene [5, 6]. One of the key phytohormones mediating drought stress responses and tolerance is abscisic acid (ABA), which regulates stomatal closure and the expression of stress response genes. Roots transmit water deficit information to the leaves, where ABA accumulates in the vasculature [7]. Drought stress is also regulated by calcium signaling, reactive oxygen species (ROS), and phytohormone translocation [8]. Furthermore, in response to drought, plants synthesize and accumulate osmolytes such as soluble carbohydrates, proteins, free amino acids, glycine betaine, and proline to increase the osmotic potential of their cells [9].

The analysis of drought stress responses using omics approaches has focused on model plants and major crops such as rice [10] and maize [11], and nonmodel species have also been studied such as *Solanum lycopersicoides* [12] and grapevine (*Vitis vinifera* [13]). So far, only two studies have considered drought stress in blackcurrant. Čereković *et al.* [3] compared irrigated and nonirrigated plants of the cultivars Narve Viking and Ben Gairn after 12 days of stress and 17 days of recovery, revealing that drought stress reduced the cumulative evapotranspiration of both cultivars during flowering due to stomatal closure and a smaller leaf area. The same authors also investigated the effect of drought on gene expression in the leaves of Ben Gairn plants [14], discovering more than 2000 differentially expressed genes (DEGs) during drought treatment based on a custom *Ribes* microarray [14]. The DEGs were found to belong to several transcription factor families, including *bZIP*, *WRKY*, *MYB*, and zinc finger. Additionally, genes associated with the cell wall and cell cycle regulation, gibberellin metabolism, the cytochrome P450 family, and the 2-oxoglutarate superfamily were identified. Although this provided first insights into the molecular basis of drought stress in blackcurrant, the specific functions of many genes remain unknown.

The blackcurrant transcriptome was analyzed in more detail by Russell *et al*. [2, 15] who identified transcriptome-based markers from leaf buds and mapped them to parental genotypes of a reference mapping population using 454 sequencing. Jarret *et al*. [16] evaluated blackcurrant fruits at different developmental stages for changes in gene expression and metabolite abundance, and used RNA-Seq data to generate a *de novo* transcriptome assembly. A transcriptome assembly based on blackcurrant fruit was published by Thole *et al.* [17], and the transcriptomic response of blackcurrant cultivar Aldoniai to blackcurrant reverse virus infection was studied by Mažeikienė *et al*. [18] and Juškytė *et al.* [19]. Although the blackcurrant chloroplast genome has been published [20], we currently lack a reference genome for the genus *Ribes* and even for the family Grossulariaceae [21].

Here we present a partially haplotype-resolved chromosome-scale genome assembly of blackcurrant (*R. nigrum* L. cv Rosenthals Langtraubige) based on a hybrid approach combining Oxford Nanopore Technologies (ONT) sequencing and PacBio HiFi long-read sequencing. We used the chromosome-scale genome, comprising eight pseudo-chromosomes, to investigate drought stress effects in Rosenthals Langtraubige leaves and roots at the transcriptomic and metabolomic levels. Our data will facilitate the development and cultivation of more drought-tolerant and climate-smart blackcurrant varieties, as well as other *Ribes* species including redcurrant and gooseberry.

## Results

### *Ribes nigrum* genome sequencing and assembly

**Figure 1:**
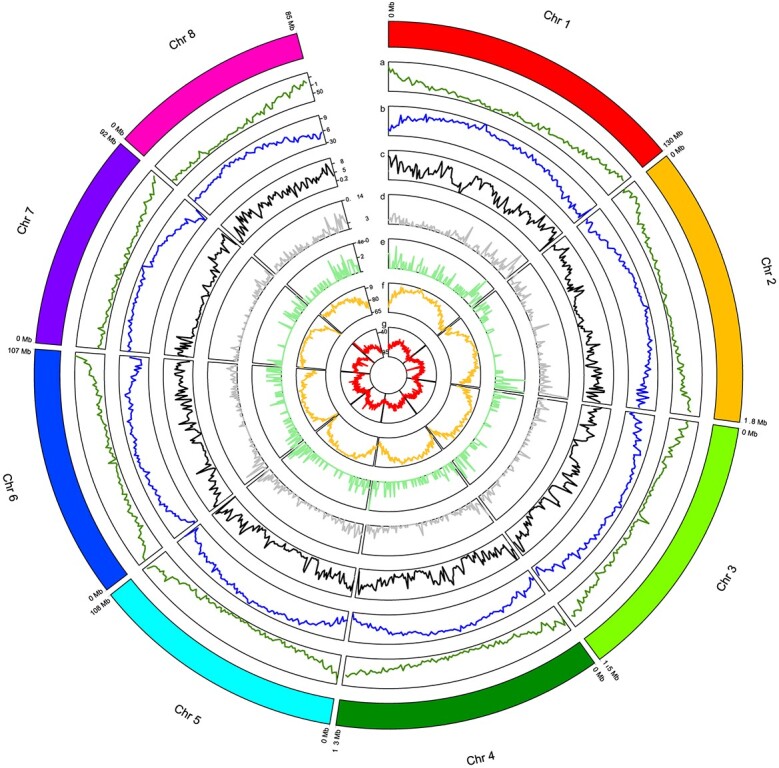
Chromosome-level characteristics of the *R. nigrum* genome. The genome is divided into eight chromosomes (Chr 1–8). (a) Gene density within 1-Mbp windows. (b) TE distribution within 1-Mbp windows. (c) Percentage of heterozygous SNP within 1-Mbp windows. (d) Percentage of heterozygous stop gained SNPs within 1-Mbp windows. (e) Percentage of heterozygous stop lost SNPs within 1-Mbp windows. (f) Percentage CpG methylation within 1-Mbp windows. (g) Percentage GC content within 1-Mbp windows.

The German cultivar Rosenthals Langtraubige was selected for the genomic analysis because the plant has a strong, spreading bushy structure, and its medium-sized, early-maturing berries have high vitamin C content. We combined PacBio HiFi and ONT nanopore sequencing with the Pore-C method for nanopore chromosome conformation capture. To this aim, we used hifiasm (UL) where we supplied the PacBio HIFI data for the initial string graph assembly and the nanopore data as ‘ultralong’ data, which hifiasm aligns to the string graph to finally produce an assembly [22]. By linking the contig sequences with the Pore-C data, we created a genome-scale assembly with a size of 871 555 738 bp (Supplemental Table S1), 99.03% of which (867 416 594 bp) was anchored to eight pseudo-chromosomes (Fig. 1, Supplemental Fig. S1).

We found that genes were more abundant at the chromosome ends but scarcer around the centromeres, where transposable elements (TEs) and CpG methylation were prevalent and the gas chromatography (GC) content was higher, a characteristic distribution found in many plant genomes (Fig. 1). The analysis of *k*-mers revealed a genome length of 800 Mb with 0.75% predicted heterozygosity, suggesting the blackcurrant genome ‘Rosenthals Langtraubige’ is relatively heterozygous (Supplemental Fig. S2).

Genome quality evaluation using the *k*-mer analysis tool Merqury [23] revealed a high consensus-based quality value (QV) of 57.50 and a *k*-mer completeness of 98.59%, indicating a very high-quality and accurate genome assembly. Telomeric sequences were identified at the beginning of chromosomes 2 and 4 and at the ends of chromosomes 3, 5, 6, and 7. A telomere-to-telomere sequence was successfully assembled for chromosome 8 (Supplemental Table S2). Furthermore, our assembly was almost gap free (0.16 Ns/100 kbp) and the long terminal repeat (LTR) assembly index (LAI), which assesses the completeness of LTR sequences [24], was 14.77, aligning closely with reference genome values for *Arabidopsis thaliana* (Arabidopsis) and other species (Table 1).

**Table 1:** Assembly statistics for the *R. nigrum* genome at the pseudo-chromosomal level

**Genome features**	***R. nigrum* genome assembly**
No. of chromosomes	8
Total length (bp)	867 416 594
GC content (%)	36.97
N per 100 kbp	0.16
*k*-mer completeness	98.60
QV	57.50
CRE(R-AQI) (%)	0.39 (96.13)
CRE(S-AQI) (%)	0.02 (97.87)
LAI	14.77

QV = quality value based on *k*-mers; LAI = LTR assembly index; R-AQI = regional assembly quality index; S-AQI = structural assembly quality index.

In accordance with these metrics, the outcomes of CRAQ quality analysis [25] demonstrated minimal clip-based regional errors (CRE = 0.39) and negligible clip-based structural errors (CSE = 0.02). The calculated average values for the regional and structural assembly quality indicators (R-AQI = 96.13 and S-AQI = 97.87) surpassed the threshold of 90, signifying a reference-level assembly quality according to Li *et al*. [25]. Furthermore, the genome was validated by benchmarking universal single-copy orthologs (BUSCO) analysis [26], resulting in a gene completeness score of 98.3% and a BUSCO duplication rate of 3.9% for the whole genome assembly. These combined results confirm the very high quality of our *de novo* genome assembly.

**Figure 2:**
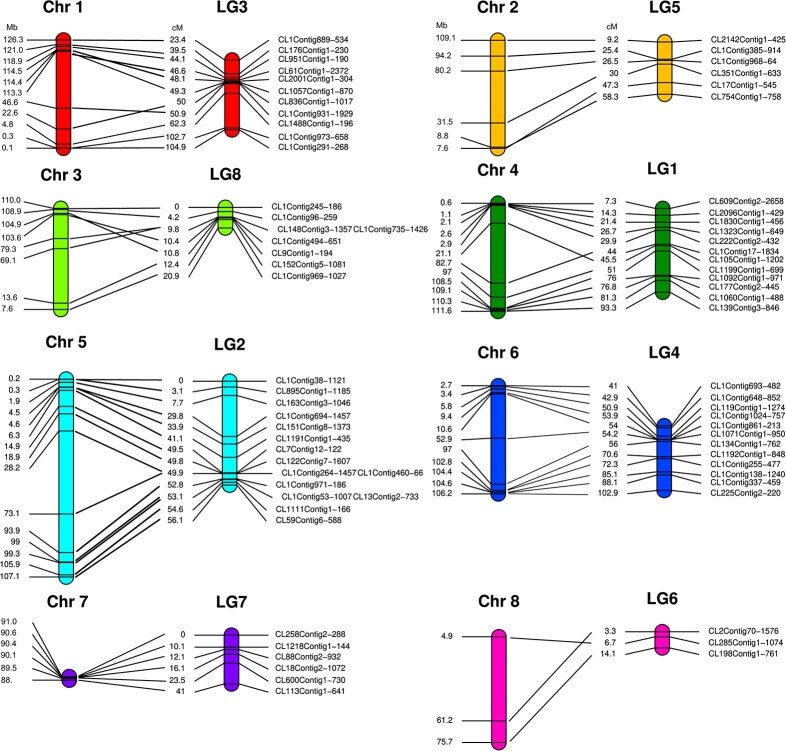
Assignment of 73 genetic markers to the *R. nigrum* reference genome. The 73 markers from the genetic map of *R. nigrum* population SCRI 9328 [2] were assigned to eight linkage groups (LG1–8), which were largely collinear with our eight pseudo-chromosomes (Chr 1–8).

We compared the *R. nigrum* genome assembly with available data by anchoring 73 single nucleotide polymorphisms (SNPs) to the linkage map of the blackcurrant SCRI 9328 population [2] (Fig. 2, Supplemental Table S3). Markers in the same linkage group were likewise found on the same chromosome in our genome assembly. Furthermore, the markers were largely collinear with the assembly. However, although markers in linkage group LG7 were collinear with our assembly, we only found them at the beginning of pseudo-chromosome 7 (Fig. 2).

TEs were identified using EDTA [24] and were found to represent 73.33% of the blackcurrant genome (Table 2). Approximately half of the identified TEs were LTRs, with Gypsy elements representing 30.35% of all TEs and Copia elements accounting for 9.03% (Table 2). Terminal inverted repeat (TIR) elements included Mutator, CACTA, PIF-Harbinger, Tc1_Marnier, hAT, and polintron. Non-LTR long interspersed nuclear elements (LINEs) accounted for only 0.27% of all TEs, whereas non-TIR helitron elements made up 2.96% of all TEs (Table 2).

**Table 2:** Repetitive DNA content of the scaffolded blackcurrant genome at the anchored pseudo-chromosome level

**Class**	**Superfamily**	**Count**	**Masked sequences (bp)**	**Masked sequences (%)**
LTR	Copia	93 001	78 309 531	9.03
	Gypsy	201 313	261 507 560	30.15
	unknown	194 422	94 960 513	10.95
TIR	CACTA	64 085	25 369 267	2.92
	Mutator	158 420	65 986 508	7.61
	PIF-Harbinger	34 197	11 702 040	1.35
	Tc1_Marnier	18 514	4 910 143	0.57
	hAT	55 797	19 738 629	2.28
	polintron	8	3314	0.00
Non-LTR	LINE	4848	2 379 098	0.27
Non-TIR	helitron	73 220	25 686 508	2.96
Repeat region		147 113	45 549 242	5.25
Total		1 044 938	636 102 353	73.33

The hybrid hifi assembly strategy, as previously outlined, also permitted the acquisition of haplotypes, with sizes of 900 Mbp for haplotype 1 and 885 Mbp for haplotype 2, and corresponding N50 values of 61 and 51 Mbp, respectively (Supplemental Table S1). Upon analyzing both haplotypes individually by total read k-mers, a *k*-mer completeness of 84.74% was achieved with a QV of 56.96 for haplotype 1, and a *k*-mer completeness of 84.84% was achieved with a QV of 58.12 for haplotype 2. This result corroborated the relatively high heterozygosity, in accordance with the 0.75% heterozygosity indicated by Genoscope analysis (Supplemental Fig. S2). Due to these results, we assessed SNPs between the two different haplotypes by calling heterozygous SNPs both using long and short read data as well as structural variants with the long read data only. This resulted in 2 176 479 SNPs and 64 644 heterozygous structural variants, respectively. Interestingly, we were able to detect somewhat lower heterozygosity in the inner parts of chromosome 7 (Fig. 1c). We further assessed the small variants and found that 1256 variants were identified that introduce premature stop codons, while 373 variants were found to result in the conversion of stop codons into nonstop codons (Fig. 1d, e; Supplemental Table S5). SNPs were functionally annotated, and variants causing stop codons showed a high enrichment in secondary metabolism [false discovery rate (FDR) < 0.05; Supplemental Table S6], while photosynthesis and phytohormone action are depleted (FDR < 0.001; Supplemental Table S6).

To make these data more accessible, we scaffolded the two haplotypes to the complete genome assembly using RagTag, enabling a simple chromosome-scale haplotype representation (Supplemental Fig. S3).

### *Ribes nigrum* genome annotation

Genome annotation, using a comprehensive pipeline that integrated *ab initio* and evidence-based approaches, predicted 42 380 gene sequences, 86.20% of which were functionally annotated using Mercator [27]. The quality of the resulting gene models was validated by BUSCO analysis, yielding a completeness score of 97.6% of the proteome (Supplemental Table S4).

### Drought stress-induced genes

**Figure 3:**
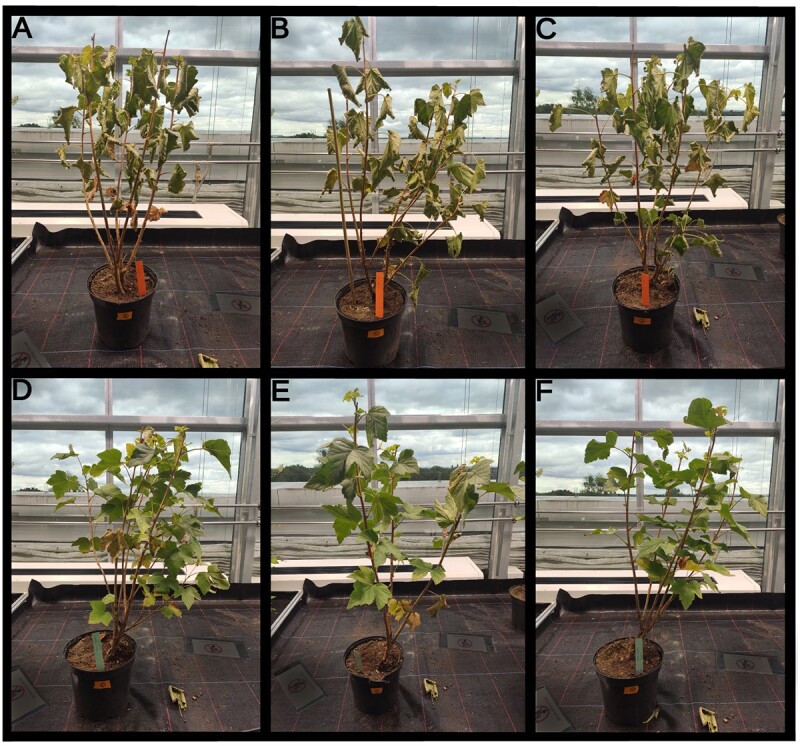
Drought stress treatment of *R. nigrum* cv Rosenthals Langtraubige plants. (A–C) Stressed plants after 4 days without water. (D–F) Control plants with continuous irrigation.

To shed light on the response of blackcurrant leaves and roots to drought stress in mature plants, water was withheld for 4 days. The treated plants showed typical external signs of a stress response, including curled and dry leaves (Fig. 3A–C). In contrast, control plants with continuous irrigation over the same period showed no change in their appearance (Fig. 3D–F).

To identify differentially expressed transcripts in leaf and root samples of adult plants, nanopore-based RNA-Seq results were used. In the leaf samples, 540 genes were identified as DEGs (FDR < 0.1), 48 of which were upregulated and 492 downregulated, as well as 8353 DEGs in the roots even with much more stringent filtering (FDR < 0.05), 3697 of which were upregulated and 4656 downregulated.

To corroborate these data, we performed a second experiment on younger plants where we again withheld water (Supplemental Fig. S4). Here, 7891 DEGs were identified in leaf samples using the FDR threshold of <0.05. Of these, 3642 genes were upregulated, while 4249 genes were downregulated.

From the 540 DEG identified in the first experiment, 80 genes (FDR < 0.05) exhibited consistent regulation patterns.

### Impact of drought stress on protein kinases

In the adult plants, we identified 243 genes encoding leucine-rich repeat (LRR) protein kinases, mainly representing families I–XV (Supplemental Table S5). For the young plants, 206 of these LRR encoding transcripts were found, showing similar expression results for most transcripts. Most LRR protein kinases were downregulated in leaves (Supplemental Table S7). However, three LRR-XV genes were slightly upregulated in leaves, and 16 LRR-XII gene transcripts were strongly downregulated (log_2_FC < −2) in leaves (Supplemental Table S7). Here, we could find 10 of these transcripts downregulated in young plants. In the roots, most LRR protein kinases were downregulated (FDR < 0.05).

Another member of the LRR family, the receptor-like cytoplasmic kinases (RLCK) multiple transcripts were of RLCK-IXb, V, VI, VIIa, VIIb, VIII, X, XI, XII, and XV found differentially expressed. Six RLCK-V transcripts were significantly downregulated in roots of adult plants (FDR < 0.01). Three transcripts of RLCK-VI (RN5G010110.1, RN1G011660.1, RN1G053710.1) were significantly downregulated in roots and have shown a strong downregulation in leaves in adult plants (Log_2_FC < −2). This expression pattern was also observed in leaf tissue in young plants (Supplemental Table S7). The downregulation was also identified for RN1G032180.1 (RLCK-X) and RN3G051940.2 (RLCK-XII) for both tissues in adult plants and was significant for roots. The RLCK-IXb gene RN4G001630.1 was significantly upregulated in roots (FDR < 0.05) and leaf tissue in young plants (FDR < 0.05) (Supplemental Table S7).

Several DEGs encoded components of the MAPK cascade. Three MAPK kinases (NPK/ANP) were upregulated in leaves but significantly downregulated in roots (FDR < 0.05) (Supplemental Table S5). In contrast, the transcript encoding MAP2K (RN3G007350.1) was slightly upregulated and MAP3K-MEKK (RN5G010030.1) was strongly downregulated (log_2_FC < −2) in leaves and RN3G007350.1 was slightly upregulated in roots (Supplemental Table S7). In young plants, these transcripts were slightly regulated but not significantly so. Ca^2+^-dependent protein kinase (CDPK) transcripts were mostly upregulated in leaves and roots, with RN1G025100.1 significantly upregulated in roots (FDR < 0.01). In contrast, two CDPK transcripts (RN1G043640.1 and RN5G008650.1) were significantly downregulated in roots and RN1G043640.1 was downregulated in leaves. One DOMAIN OF UNKNOWN FUNCTION 26 (DUF26) transcript was significantly (FDR < 0.05) upregulated in roots and two others were significantly downregulated (Supplemental Table S7). In young plants, the transcript RN8G019550.2 (DUF26) was significantly upregulated (FDR < 0.01), which showed the same expression in leaf and root tissue of adult plants.

Five transcripts encoding proline-rich extensin-like receptor kinases (PERKs) were downregulated in leaves, including RN4G048640.1, RN7G025360.1, and RN7G034400.1, which were also significantly downregulated in roots (FDR < 0.01). In contrast, RN3G015760.1 was significantly upregulated in roots (FDR < 0.01) (Supplemental Table S7). Transcripts in young plants in leaf tissue showed the same expression pattern as roots in adult plants, and RN3G015760.1 was also significantly upregulated (FDR < 0.01).

### Transcription factors and metabolism

Transcripts encoding *AP2/ERF*, *bZIP*, *CBF/DREB1*, *DREB2*, *C2H2*, *HD-ZIP*, *R2R3-MYB*, *WRKY*, *HD-ZIP*, *bHLH*, and *NAC* transcription factors were differentially expressed in both tissues of adult plants. Transcripts associated with carotenoid and ABA metabolism, including those encoding PYL/RCAR and abscisic aldehyde oxidase, were generally downregulated, and the PYL/RCAR transcripts RN3G031060.1, RN4G014070.1, and RN4G030230.1 were significantly (FDR < 0.01) downregulated in the roots (Supplemental Table S7). In young plants in leaf tissue, RN3G031060.1 showed the same significant downregulation (FDR < 0.001) as in roots in adult plants. Several transcripts encoding SnRK1 and SnRK2 SNF1-related protein kinases were modulated in both tissues in adult plants and also in leaf tissue in young plants, with significant differential expression in the roots. Several transcripts associated with cell wall regulation were identified in the leaves. Genes encoding *p*-*coumaroyl shikimate/quinate 3-hydroxylase* were modulated in leaves and roots (Supplemental Table S5). RN6G009060.1, encoding hydroxyproline-*O*-galactosyltransferase (GALT29), was significantly downregulated in roots and slightly downregulated in leaves. Transcripts encoding caffeoyl-CoA 3-*O*-methyltransferase were significantly downregulated in roots but were expressed at minimal levels in leaves (Supplemental Table S7).

### Relative expression of drought stress induced through qRT-PCR

To validate the results of drought-stress induced genes, a total of 13 DEGs, comprising six upregulated and seven downregulated genes in leaf tissue of adult plants, were selected for qRT-PCR. The analysis was conducted using actin and eIF4A as endogenous controls. The results indicated that the selected genes exhibited comparable gene expression fold changes (FCs) between quantitative qRT-PCR and computational analyses in both leaf and root tissues of mature plants (Supplemental Fig. S5B). Additionally, qRT-PCR was conducted for the same genes, excluding 4HbD and WRKY, which could not be reliably quantified in the leaf tissue of young plants. Of these 11 genes, the expression of eight genes were consistent with those observed in the leaf tissue of drought-stressed adult plants, further validating the selected candidate genes involved in the *R. nigrum* response to drought stress (Supplemental Fig. S5A, B).

### Metabolite changes due to drought stress

Next, we compared the abundance of 60 primary metabolites in response to drought stress in leaves and roots of mature plants. Several were barely affected, but 14 leaf metabolites became significantly more abundant under stress (log_2_FC > 1, FDR < 0.05). Most of these metabolites were amino acids and their derivatives such as threonine, valine, proline, isoleucine, methionine, glutamine, glutamic acid, tryptophan, phenylalanine, alanine, and 4-aminobutanoic acid (GABA) (Table 3, Supplemental Tables S6 and S8). Sugars such as mannose, sugar derivatives such as galactinol, and the organic acid quinic acid also accumulated in response to drought, whereas citric acid was the only metabolite that was significantly depleted.

**Table 3:** Metabolites in blackcurrant leaf tissue that change in abundance in response to drought stress

$\includegraphics{\bwartpath uhae313t3}$

Upregulation (red) and downregulation (blue) is shown according to the log_2_FC of the genes. Significance levels of adjusted *P* values (FDR) are shown in bold.

Few metabolic changes were observed in the roots. However, mannose and isoleucine accumulated in response to drought stress (log_2_FC > 1.3), whereas β-alanine was depleted (log_2_FC < −1.3). We also observed a slight increase in the levels of valine, isoleucine, and methionine (log_2_FC > 0.5), and a slight decrease in the levels of citric acid (log_2_FC < −0.5) (Supplemental Fig. S7). Although these metabolites showed minor changes according to their log_2_FC values, the adjusted *P* value suggested that the changes were not significant. In the leaf tissue of young plants, similar patterns were observed for valine, isoleucine, proline, threonine, GABA, and phenylalanine compared to the leaf tissue of adult plants. Additionally, valine, proline, GABA, and phenylalanine exhibited significant upregulation (FDR > 0.05), while alanine, serine, and glutamic acid showed a slight downregulation (Supplemental Table S9).

### Connecting DEGs with metabolites in response to drought stress

**Figure 4:**
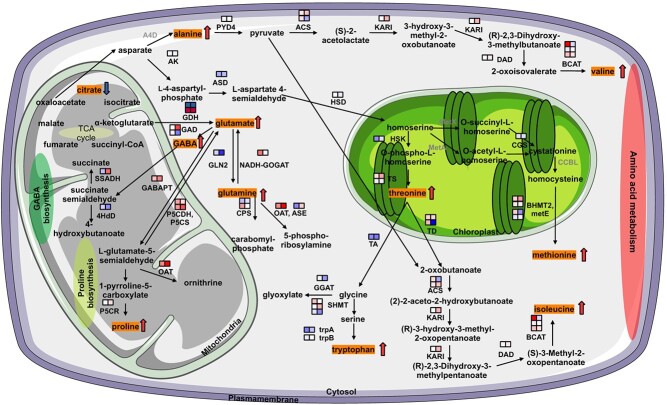
The connection between DEGs and metabolic pathways that significantly alter metabolite levels under drought stress in the leaves and roots of *R. nigrum*. DEGs encoding enzymes (annotated using MapMan) are aligned with specific metabolic reactions (absent = bold gray). Upregulated genes are denoted by red squares and downregulated genes by blue squares, with squares on the left and right indicating leaves and roots, respectively. Multiple rows of squares denote multiple genes with the same enzymatic functions (log_2_FC of DEGs and FDRs listed in Supplemental Table S2). Metabolites differing in abundance (Table 3 and Supplemental Tables S1 and S2) under drought stress are highlighted in orange, with accumulation shown as red arrows and depletion as blue arrows. Abbreviations: 4HbD = bifunctional γ-hydroxybutyrate dehydrogenase; ACLY = ATP citrate (pro-S)-ligase; ACO = aconitase; ACS = acetolactate synthase; AK = aspartate kinase; ASAT = aspartate transaminase; ASD = aspartate-semialdehyde dehydrogenase; ASE = amidophosphoribosyltransferase; A4D = aspartate 4-decarboxylase; BCAT = branched-chain-amino-acid transaminase; BHMT2 = homocysteine S-methyltransferase; CCBL = kynurenine-oxoglutarate transaminase; CGS = cystathione-γ-synthase; CPS = large subunit of carbamoyl phosphate synthetase heterodimer; DAD = dihydroxy-acid dehydratase; GABAPT = GABA pyruvate transaminase; GAD = glutamate decarboxylase; GAT = GABA transporter; GDH = glutamate dehydrogenase; GGAT = glycine transaminase; GLN1 = cytosolic glutamine synthetase; GLN2 = plastidial glutamine synthetase; HSD = homoserine dehydrogenase; HSK = homoserine kinase; IDH3 = isocitrate dehydrogenase heterodimer subunit 1; KARI = ketol-acid reductoisomerase; metA = homoserine O-succinyltransferase; metE = 5-methyltetrahydropteroyltriglutamate homocysteine methyltransferase; metX = homoserine O-acetyltransferase; NADH-GOGAT = NADH-dependent glutamate synthase; OAT = ornithine-γ-aminotransferase; P5CDH = [1]Δ-pyrroline-carboxylate dehydrogenase; P5CR = pyrroline-carboxylate-dehydrogenase; P5CS = [1]Δ-pyrroline-carboxylate synthase; PYD4 = alanine aminotransferase; SHMT = serine hydroxymethyltransferase; SSADH = succinate-semialdehyde dehydrogenase (NAD+); TA = threonine aldolase; TD = threonine dehydratase; trpA = subunit α of tryptophan synthase complex; trpB = subunit β of tryptophan synthase complex; TS = threonine synthase.

The integration of data from the analysis of DEGs and metabolites revealed associations involving the tricarboxylic acid (TCA) cycle, GABA biosynthesis, cysteine and methionine metabolism, proline metabolism, alanine and aspartate metabolism, as well as branched-chain amino acid metabolism. We also found associations between groups of altered metabolites in leaves (FDR < 0.05) and between groups of DEGs in both tissues (Fig. 4).

In the context of the TCA cycle and the depletion of citric acid, all related transcripts in leaves in adult plants were strongly upregulated (Supplemental Table S8) with the exception of *ACLY* (RN7G026170.1), which was downregulated in leaves and roots. In young plants, this transcript showed a similar significant downregulation (FDR < 0.01). GABA-associated genes were mainly upregulated in both tissues in adult and young plants. Notably, one gene encoding a bifunctional γ-hydroxybutyrate dehydrogenase (RN1G038820.1) was significantly downregulated in roots, while another (RN3G012700.1) showed no significant differential expression in roots. *Glutamate decarboxylase* genes were significantly upregulated (RN8G004390.1) or downregulated (RN6G037990.1) in roots but were not expressed at significant levels in leaves. The *succinate-semialdehyde dehydrogenase* gene RN8G041850.1 was significantly upregulated in roots but showed no expression in young plants in leaf tissue (Supplemental Table S8). Genes involved with alanine, aspartate, and glutamate metabolism were generally downregulated in both tissues in adult and young plants (except *ASAT*, demonstrated upregulation). Furthermore, genes encoding *CPS* and *NADH-GOGAT* were observed to be upregulated in leaves, while a threonine aldolase gene demonstrated a significant downregulation in the roots (Supplemental Table S8). All transcripts associated with valine, leucine, and isoleucine metabolism showed significantly upregulated in adult plants. One *threonine dehydratase* gene (RN6G010130.1) exhibited a marked decrease in roots (Supplemental Table S8). In young plants, most of these transcripts were downregulated except RN4G055970.1 and RN2G016250.1, which were upregulated (Supplemental Table S8).

**Figure 5:**
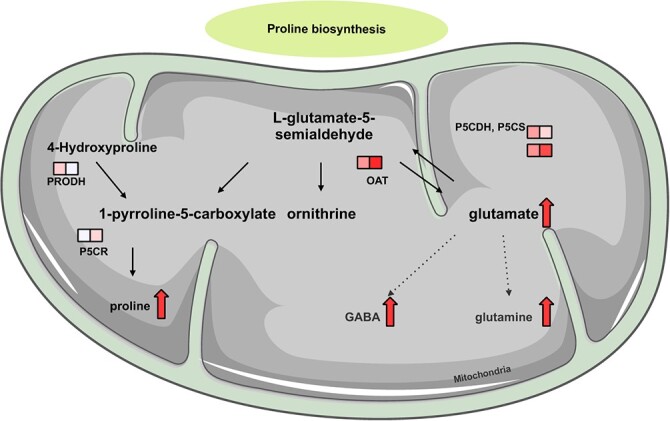
Proline biosynthesis metabolites with significantly altered levels under drought stress in the leaves of *R. nigrum*. DEGs (Table 4) encoding enzymes (annotated using MapMan) are aligned with specific metabolic reactions. Upregulated genes are denoted by red squares and downregulated genes by blue squares, with squares on the left and right indicating leaves and roots, respectively. Multiple rows of squares denote multiple genes with the same enzymatic functions. Metabolites that accumulate under drought stress (Table 4 and Supplemental Tables S1 and S2) are highlighted with a red arrow. Abbreviations: OAT = ornithine-γ-aminotransferase; P5CDH = [1]Δ-pyrroline-carboxylate dehydrogenase; P5CR = pyrroline-carboxylate dehydrogenase; P5CS = [1]Δ-pyrroline-carboxylate synthase; PRODH = proline dehydrogenase.

Transcripts in adult plants involved in proline metabolism were mostly upregulated in leaves, although several glutamate dehydrogenase transcripts were downregulated and the same genes were significantly (FDR < 0.05) downregulated in roots (Table 4, Fig. 5). These results were also found in leaf tissue in young plants, except for some glutamate dehydrogenase, which have shown no expression. For transcripts of P5CDH (RN8G016110.1), OAT (RN2G017990.2), P5CR (RN2G007260.1), and P5CS (RN4G032560.1), expression in young plants was similarly regulated as in leaf tissue of young plants, while OAT, P5CR, and P5CS were significantly differentially expressed (FDR < 0.05). Transcripts encoding ornithine-γ-aminotransferase and two [1]Δ-pyrroline-carboxylate synthases were significantly upregulated in roots of adult plants (FDR < 0.01).

**Table 4:** Differentially expressed transcripts involved in proline metabolism and their MapMan annotations

$\includegraphics{\bwartpath uhae313t4}$

Upregulation (red) and downregulation (blue) reflect the log_2_FC values, with significance (FDR adjusted *P* values) shown in bold.

## Discussion

### *Ribes nigrum* genome assembly

We have generated a blackcurrant genome assembly at the pseudo-chromosomal level, the first published genome representing the genus *Ribes* or the family Grossulariaceae, and have identified multiple genes associated with drought stress as well as related changes in metabolites. The total size of the anchored assembly was ~871 Mbp, which is close to the estimated genome size of ~1 Gbp for the genus *Ribes* [21] and in line with the Genoscope estimation of 750 Mbp (Supplemental Fig. S2). We used a hybrid approach that integrates ONT long reads and precise HiFi PacBio data directly in hifiasm, as well as Pore-C ONT contact data, thus coupling long reads directly to multiway chromatin contacts without amplification [28]. This allowed the construction of pseudo-chromosomal scaffolds representing eight chromosomes, building on the complementarity of simplex ONT and precise HiFi reads [29, 30]. Our data indicate that blackcurrant is a diploid genome with a basic chromosome number of 8, in line with other *Ribes* species [21, 31]. Given the relatively high heterozygosity of cv Rosenthals Langtraubige, we generated contiguous individual assemblies for haplotypes 1 and 2, with sizes of 900 and 885 Mbp, respectively, and scaffolded these against the haploid assembly using RagTag. While this later step introduces several switching errors, it might provide a useful representation for breeding and analysis purposes. Structural variant analysis identified a substantial number of stop codons across the genome, suggesting a high frequency of gene disruption and an elevated mutational load.

The quality of the genome assembly was checked using the LAI, which estimates completeness by calculating the percentage of intact LTR retrotransposon sequences [32]. The LAI of the blackcurrant genome was 14.77, similar to the score of 14.9 reported for Arabidopsis [24, 33]. Long-read sequencing methods like ONT and PacBio typically yield LAI scores >10, which designates a genome of reference quality [34]. This was supported by the assessment of errors using CRAQ, which revealed an R-AQI of 96.21% and an S-AQI of 98.64%, with scores >90% indicating reference quality [25]. The AQI scores for the blackcurrant genome were comparable to those of the PacBio complete long reads (CLR) rice haplotype MH63 (R-AQI = 96.25; S-AQI = 94.96) (https://www.ncbi.nlm.nih.gov/datasets/genome/GCA_001623365.2 [25]) and the PacBio CLR rice haplotype R498 (R-AQI = 96.96; S-AQI = 98.22) [25, 35] at the chromosomal level. Our results surpassed the existing ONT assemblies of *Solanum pennellii* (R-AQI ≤ 95.57; S-AQI = 91.47) and Arabidopsis KM74 (R-AQI = 85.95; S-AQI = 93.34), as well as the PacBio CLR Arabidopsis KM74 assembly [33] at the contig level (R-AQI = 95.57; S-AQI = 96.29) (Table 1 [25]). These distinctions probably reflect variations in sequencing methods and TE identification. Notably, PacBio HiFi error rates are generally lower than those of simplex ONT reads, yielding more accurate assemblies. Our R-AQI values align closely with the PacBio reference genomes of rice and Arabidopsis.

The QV of our assembly assessed using Merqury was 57.49, which corresponds to 99.999% accuracy [23]. This improves upon the PacBio MH63 gap-free rice reference genome [36], which achieved a QV of 55.05, and the chromosome-level assembly based on PacBio and Hi-C data of the berry *Fragaria pentaphylla* [37], which achieved a QV of 54.76. Furthermore, the *k*-mer completeness of our genome was 98.59, which is higher than the PacBio and Hi-C *de novo* assemblies of *F. pentaphylla* and *Arctostaphylos glauca*, with *k*-mer completeness values of 84.71 and 74.39, respectively [37, 38], and similar to the 98.87 score assigned to the PacBio haplotype-resolved assembly of *Coriaria nepalensis* [39]. To make our assembly useful for quantitative trait loci (QTL) studies, we mapped SNP marker sequences from the linkage map of population SCRI 9328 and MP7 [2]. Genetic markers in specific linkage groups were also identified on corresponding chromosomes in our assembly. This demonstrates concordance between the genetic and physical maps and shows that our assembly is accurate and can be used to compare legacy data with our genome assembly.

The high quality of our sequence assembly was also confirmed by the BUSCO completeness score of 98.3%. This surpasses the scores of chromosomal-level genomes in the Saxifragaceae family, which is part of the Saxifragaceae alliance alongside the Grossulariaceae [40]. Three Saxifragaceae genomes have been published at the chromosomal level. The largest (12 Gbp, five chromosomes) is *Paeonia ostii*, which achieved a BUSCO completeness score of 94.4%, and the smallest (412 Mbp) is *Tiarella polyphylla*, which achieved a BUSCO completeness score of 97.31% [41, 42]. The *Cercidiphyllum japonicum* genome (719 Mbp, 19 chromosomes) achieved a score of 94.1%. The *T. polyphylla* assembly is based on a combination of Illumina short reads, ONT long reads, and Hi-C data, whereas the assembly of the *C. japonicum* genome is based on PacBio long reads, Illumina short reads, and Hi-C data. The higher BUSCO completeness score of our assembly may reflect the synergistic integration of PacBio and ONT reads with ONT Pore-C data. Our high BUSCO completeness score also surpasses the 97%–98% range reported for other berry crops, such as the blueberry wild relative *Vaccinium darrowii* with a BUSCO score of 97.5% [43], and raspberry (*Rubus idaeus*) with a BUSCO score of 91.3% [44]. These genomes were assembled using PacBio, Hi-C, and Illumina data, explaining the slightly lower BUSCO scores compared to our assembly. Our assembly, anchored on eight pseudo-chromosomes, successfully mapped 99% of the genome, whereas the comparable *C. japonicum* genome only anchored 90.18% on 19 pseudo-chromosomes [45]. Our annotation allowed the prediction of 42 380 genes with a BUSCO completeness score for the transcriptome of 96.8%. In comparison, Thole *et al.* [17] assembled 145 906 transcripts (N50 = 1480 bp) with a read mapping rate of 90.4% for cv Ben Hope. Another 40 225 genes were predicted by microarray analysis, but the data are not publicly available for comparison [14].

### DEGs in leaf and root tissue during drought stress

#### Protein kinases

The genome assembly and annotated transcriptome were used to identify DEGs in leaf and root samples from drought-stressed plants and controls. During drought stress, plants close their stomata to minimize water loss, promote deeper root growth to enhance water acquisition, and shed leaves to reduce transpiration. The perception of drought stress involves the transduction of signals via receptor-like kinases [46]. Accordingly, we identified many DEGs encoding LRR kinases, most of which were significantly downregulated in roots and leaves under drought conditions. The LRR-III gene RN5G011100.1 was downregulated in both leaf and root tissues, a finding further validated by qRT-PCR. This supports the hypothesis that LRR-III plays a role in the drought stress response of *R. nigrum*. Besides that, one gene annotated as a member of the subfamily of LRR-XV was upregulated in leaves and significantly downregulated in roots. Ksouri *et al*. [47] previously observed a similar trend in *Prunus persica* rootstocks, noting the downregulation of LRR kinase genes particularly in leaves. To date, LRR and RLK (receptor-like kinase) proteins are known to play crucial roles in the perception of environmental signals and the initiation of responses to various biotic and abiotic stresses. However, LRR-III has not been directly associated with drought stress response in plants [48].

We also identified DEGs encoding RLCKs that form complexes responsible for intracellular signaling to control plant growth, development, and stress responses [49]. RLCK-VII in particular is closely associated with plant growth and development, suggesting its significant role in signal transduction [50]. Several Arabidopsis RLCK-VII proteins are involved in brassinosteroid signaling and root hair growth as well as root development and stomatal defenses (Mara-Garcia *et al*., 2004). Furthermore, RLCK-VIII is associated with oxidative stress signaling [51, 52]. Members of the RLCK-XII family, such as BSK1, are essential for flg22-induced maximal ROS production, a key component of immune signalin [51, 53]. Additionally, RKS1 and the NLR (nucleotide-binding LRR) protein ZAR1 work together to initiate immune responses, contributing to plant defense mechanisms in Arabidopsis [54, 55]. We observed the modulation of RLCK genes representing classes VII, VIII, and XII in both tissues in adult and young plants. Furthermore, we detected DEGs encoding RLCK classes V, VI, X, XI, and XII that have not been extensively characterized but appear to be involved in responses to drought stress in both tissues in adult, and were strongly downregulated in the roots. Additionally, we found these genes in leaf tissue in young plants as well, validating our findings of RLCK genes under drought stress in *R. nigrum* under drought stress.

We identified six PERKs expressed in leaf and root tissues, one of which was upregulated in roots but downregulated in leaves. Arabidopsis PERK4 is predicted to be a key regulator of Ca^2+^ signaling, contributing to the production of ABA in roots [56]. Furthermore, several ZmPERK genes are induced in response to stress [57]. The cysteine-rich repeat receptor-like kinase family (CRK) is typically characterized by the presence of two DUF26 protein interaction domains involved in sensing of environmental signals and the regulation of plant development [58, 59].

#### Hormone-related genes

Pyrabactin resistance (PYR), pyrabactin like (PYL), and regulatory component of ABA receptors (RCAR) are major negative regulators of the ABA signaling cascade if no ABA is available [60]. We found that most PYL/RCAR components were downregulated in leaves and roots of adult plants. This result was also found in young plants in leaf tissue for one PYL/RCAR component. These proteins form a complex with SnRK1, SnRK2, and PP2C. ABA can bind to PYR/PYL/RCAR and activate the SnRK1, SnRK2, and SnRK3 kinases. Several SnRK3 (data not shown) and SnRK2 genes were upregulated in our study, and a gene encoding the regulatory FCS-like zinc finger (FLZ) of the SnRK1 complex was downregulated, suggesting the ABA pathway is activated in response to drought stress. The involvement of this pathway and the corresponding genes in abiotic stress responses has already been shown in Arabidopsis [61], barley [62], pea [63], and fava bean [64]. The same genes also regulate development, growth and stomatal movement [65].

#### Transcription factors

Drought stress influenced the expression of many stress-responsive genes encoding transcription factors in leaves and/or roots. The ERF-I, II, IV, IX, VI, VIII, X, and DREB1/2 genes were upregulated in both tissues in adult and young plants, and were previously shown to regulate drought stress responses [66]. CBF/DREB1 and DREB2 mediate drought tolerance in rice, soybean, and Arabidopsis [66, 67]. We likewise found that most CBF/DREB1 and DREB2 genes were upregulated under drought stress. Čereković *et al.* [14] showed that three bZIP transcription factors were modulated in *R. nigrum* leaves under drought stress, which were names according to similar Arabidopsis genes: *AT3G58120* (upregulated), as well as *AT5G24800* and *AT4G35900* (downregulated). We found that multiple bZIP genes were upregulated in both tissues. The difference between the studies may reflect the use of different cultivars and/or annotated reference genomes. The IRE1/bZIP60 gene and corresponding regulatory mediators were strongly downregulated in roots. IRE1/bZIP60 is part of the unfolded protein response.

*R2R3-MYB, bHLH, WRKY, NAC, zinc finger (C2H2)*, and *HD-ZIP* transcription factors are also involved in drought stress responses [68–73]. R2R3-MYB and bHLH proteins have been linked to the anthocyanin pathway and ABA-dependent regulation in *R. nigrum* under drought stress [1, 14, 74, 75]. Čereković *et al.* [14] showed that MYB genes were downregulated under drought stress, which is consistent with our results for *R. nigrum* R2R3-MYB genes. Furthermore, we found that WRKY, NAC, C2H2, and HD-ZIP genes were modulated in *R. nigrum* leaves and roots of adult and young plants. The *R. nigrum* WRKY33 gene was reported to be induced in leaves under drought stress [3] and mediates the drought stress response in *Caragana korshinskii* [71]. Plants expressing CkWRKY33 were more likely to survive a period of drought and featured higher levels of soluble sugar, proline, and peroxidase activity. We found that several NAC genes were differentially expressed in *R. nigrum* leaves and with highly significant differences in the roots, in agreement with previous findings [76, 77]. NAC proteins play a key role in drought responses, activating stress-responsive genes or enhancing drought resistance in transgenic plants [76, 77]. Čereković *et al.* [3] found that several C2H2 genes were downregulated in *C. korshinskii* leaves and roots, whereas we detected both upregulated and downregulated C2H2 genes in both tissues. C2H2 transcription factors influence ROS scavenging in rice by regulating the expression of genes encoding antioxidants and the synthesis of osmoprotectants such as proline and soluble sugars [68, 78, 79]. They are also involved in ABA signaling by modulating genes associated with stomatal closure and root development in response to drought [79]. C2H2 transcription factors also regulate the MAPK signaling pathway [80–82]. Finally, we observed the modulation of HD-ZIP genes in both tissues. The corresponding transcription factors are known to regulate growth, development, drought stress responses, ABA signaling, and stomatal closure [83–86].

### DEGs that influence the abundance of metabolites

We identified multiple DEGs related to ABA, GABA, and proline biosynthesis, as well as general amino acid metabolism, and we also found that several of the corresponding metabolites were affected. Interestingly, most of these metabolic differences were restricted to the leaves, whereas previous studies have shown changes in roots too, for example, in peanut and various Triticeae species (Hu *et al*., 2016 [87, 88]). The differences between our study and the previous reports may be species dependent or may reflect the relatively short period of drought stress we applied.

The main metabolic differences we observed between stressed leaves and controls involved the accumulation of amino acids, which serve as osmolytes, antioxidants, signaling mediators, and sources of energy [89, 90]. Proline and GABA accumulated in the stressed blackcurrant leaves in adult and young plants. Proline is an osmolyte and chaperone that accumulates to high levels in many dehydrated plants [76, 91–94]. GABA increases leaf turgor and osmolyte synthesis, regulates antioxidant levels, and increases water use efficiency and drought tolerance by influencing guard cell behavior [95]. Genes associated with these pathways were also found among the DEGs (Table 2, Fig. 3). Glutamine is another osmoprotectant that accumulates under drought stress [89]. It is synthesized from aspartate and converted to glutamate, which are involved in the transduction of Ca^2+^, ROS, and electric signals, and it is also a precursor of proline, asparagine, arginine, and GABA [74, 96–99]. These metabolites, which play a key role in nitrogen metabolism, also accumulated in our drought-stressed leaves, and the corresponding regulatory genes were also differentially expressed (Table 2 and Fig. 3).

Citric acid is an organic acid within the TCA cycle that can be utilized for amino acid or GABA biosynthesis via the production of glutamate [100]. Drought stress can disrupt the TCA cycle by reducing oxygen availability and respiration, leading to alterations in citric acid levels [101]. We found that citric acid was depleted in stressed leaves, in agreement with previous studies (Ashraf *et al*., 2018). Quinic acid, which was slightly elevated in the stressed leaves, is known to be involved in drought stress responses, acting as a precursor for the synthesis of phenolic compounds and osmolytes [73, 102].

The amino acids threonine, methionine, isoleucine, and valine also accumulated under drought stress. Threonine and methionine are precursors of isoleucine and valine, which accumulate as a response to osmotic stress in Arabidopsis and other plants [91, 103–105]. These amino acids regulate osmosis and reduce damage caused by ROS (Singh *et al*., 2015; Bohnert and Jensen, 1996). The OsDIAT gene encodes a regulator of branched-chain amino acid accumulation in rice [106]. In our study, the corresponding gene RN4G055970.1 (*BCAT*) was upregulated in both tissues of adult plants and in leaf tissue of young plants, which may underpin the accumulation of isoleucine and valine (Table 2, Fig. 4).

The aromatic acids phenylalanine and tryptophan, which accumulated in our stressed plants, are precursors of secondary metabolites such as indole acetate, lignin (via the shikimate pathway), and flavonoids, all of which play important roles in drought stress tolerance [107] and plant growth [108]. Phenylalanine levels were previously shown to increase during dehydration and decrease after dehydration in *Barbacenia purpurea* [107]. Tryptophan is the main precursor of auxin, which regulates vacuolar osmotic pressure and the translocation of metabolites, thus improving osmotic balance and growth under drought stress [109]. Tryptophan has been reported to accumulate under drought stress in other plants, which is consistent with our results [110].

The accumulation of sugars in plants during drought stress is an important adaptive strategy that supports survival during drought and recovery afterward [111]. In our study, the galactinol content of the leaves increased under drought stress, which has been previously reported in other plants [112, 113]. Sucrose, glucose, and fructose also contribute to drought stress tolerance, but we did not observe the accumulation of these soluble sugars in blackcurrant. As discussed above, this may reflect the relatively short duration of stress in our experiments, which may have been insufficient for the breakdown of storage carbohydrates into monosaccharides and disaccharides. In the context of sugar metabolism, we identified an arabinogalactan protein, GALT29/GALT29A, which was significantly downregulated across all tissues in both young and adult plants under drought stress (Supplemental Fig. S9). In *A. thaliana*, AtGALT29A encodes a putative glycosyltransferase that belongs to the carbohydrate-active enzyme family GT29. AtGALT29A exhibits β-1,6-galactosyltransferase activity, elongating β-1,6-galactan side chains and forming 6-Gal branches on the β-1,3-galactan backbone of arabinogalactan proteins [114]. So far, GALT29A has not been described as being involved in the drought stress response. However, our results strongly suggest that it plays a role in both leaf and root tissues of *R. nigrum* under drought conditions.

## Conclusion

We have constructed a blackcurrant (*R. nigrum*) haplotype-resolved *de novo* genome assembly at the pseudo-chromosomal level using a combination of ONT long reads, Illumina short reads, and ONT Pore-C sequencing data. The resulting high-quality assembly (confirmed by BUSCO scores, *k*-mer completeness, and AQI values) is the first for the genus *Ribes* and indeed for the family Grossulariaceae. Transcriptome analysis revealed many DEGs involved in drought stress mechanisms, which were connected to changes in metabolite levels, particularly involving the TCA cycle, amino acid metabolism, and the biosynthesis of proline and GABA. These integrated findings enhance our understanding of *R. nigrum* stress-related genes and metabolites. These findings were observed in both adult and young plants, confirming the validity of our results regarding the drought stress response of *R. nigrum* in leaf tissues. However, more specific analysis (targeted metabolomics) is needed to establish precise relationships spanning the genome, transcriptome, and metabolome. The annotated blackcurrant genome presented in this study lays the foundation for further research to determine the molecular basis of drought stress responses in this important fruit crop. Our results can also be combined with existing genetic resources such as quantitative trait loci to facilitate marker-assisted selection and genetic engineering for the development of improved blackcurrant cultivars.

## Materials and methods

### Plant material

For DNA sequencing, young blackcurrant (*R. nigrum* cv Rosenthals Langtraubige) leaves were collected from a single adult plant and flash frozen in liquid nitrogen. For the drought stress experiments, plants in 3-l pots were irrigated with 150 ml of water per day (control) or water was withheld for 4 days (stress treatment). Root and leaf samples (three replicates per plant) were taken the morning after the last treatment day. Blackcurrant cv Tihope plants were grown under field conditions in Poland. Samples were taken at different developmental stages: flower initiation, dormancy induction, and dormancy release. Bud samples were collected at nine different time points, whereas leaf samples were taken at three time points during flower initiation. The samples were used for RNA deep sequencing on the Illumina HiSeq 2500 platform. Blackcurrant RNA-Seq datasets of cv Ben Gairn were generated from 20 bud samples on the same sequencing platform.

Stem cuttings of *R. nigrum* cv. ‘Rosenthals Langtraubige’ were cultivated in 500-ml pots for 5 weeks under controlled greenhouse conditions. For the drought stress experiment, plants were initially irrigated until the soil reached full water-holding capacity. Subsequently, plants in the control group were watered to maintain 70% of pot weight based on water loss, while those in the stress treatment group received no water for a period of 9 days. Leaf samples were collected the morning following the final day of treatment. RNA was extracted as described below, and samples were used for RNA deep sequencing on the Illumina HiSeq 2500 platform.

### DNA library construction and sequencing

DNA was extracted from 1 g of frozen leaf material using the NucleoBond HMW DNA Kit (Machery Nagel, Thermo Fisher Scientific, Waltham, MA, USA). Short fragments were separated using the Circulomics short read eliminator (SRE XL) Kit (Pacific Biosciences, Melno Park, CA, USA) with a threshold of >40 kbp. DNA degradation and contamination were monitored by 1% agarose gel electrophoresis. DNA purity was checked using a NanoDrop spectrophotometer (Thermo Fisher Scientific), and the DNA concentration was determined using the Qubit DNA Assay Kit and a Qubit fluorometer (Thermo Fisher Scientific). Filtered long fragments were used to prepare ONT libraries for sequencing (Qubit DNA Assay Kit and a Qubit fluorometer). We used the standard ONT protocol for the SQK-LSK112 Kit. Genomic DNA fragments were repaired and 3′-adenylated using the NEBNext FFPE DNA Repair Mix and the NEBNext Ultra II End Repair/A-Tailing Module (New England Biolabs/NEB, Ipswich, MA, USA). Sequencing adapters provided by ONT were then ligated using the NEBNext Quick Ligation Module (NEB). After purification with AMPure XP beads (Beckmann Coulter, Brea, CA, USA), libraries were loaded onto primed 10.4.1 Spot-On Flow Cells and sequenced using a PromethION device (ONT) with 72-h runs. Cells were flushed and reloaded after 18 h. Basecalling was achieved using guppy v6.1.1 (ONT). PacBio sequencing of the ONT long-read sequences was carried out by Genohub (Brigham Young University, Provo, UT, USA). Filtered short-read sequences were sequenced by Genewiz (Leipzig, Germany).

### cDNA library construction and sequencing

Total RNA was extracted from leaves and roots of adult plants of treated and control plants using the RNeasy Plant Mini Kit (Qiagen, Hilden, Germany) according to the manufacturer’s recommendations, with minor changes. Briefly, 50 mg of frozen tissue from each plant was lysed (1.5 M NaCl, 2% CTAB, 30 mM EDTA pH 8, 100 mM Tris-Cl, 2% β-mercaptoethanol in RNase-free water) for 10 min at 70°C. Further extraction and on-column digestion with DNase I was carried out according to Qiagen recommendations. RNA quality was monitored as described above for DNA. For cDNA sequencing, 200 ng of RNA per sample was used in a strand-switching step followed by cDNA amplification by PCR using primers containing 5′ tags according to the ONT protocol for cDNA PCR sequencing (SQK-PCS111). We then added rapid sequencing adapters and loaded 25–55 ng onto primed 9.4.1 Spot-On Flow Cells for sequencing using a PromethION device.

### Analysis of gene expression of selected drought-responsive genes by qRT-PCR

Based on the results of the RNA-Seq analysis, genes responsive to the drought stress treatment were selected for validation of their expression in leaves and roots of adult plants and leaves in young plants (Supplemental Table S11). Actin and eIF4A [19, 115] were selected for normalization. For cDNA synthesis, 1 μg of RNA was reverse transcribed into cDNA using iScript cDNA Synthesis kit (Bio-Rad) in a 20-μl reaction following the manufacturer's instructions. Quantitative real-time PCR of selected genes was conducted with a CFX Opus 384 Real-Time PCR System (Bio-Rad). The amplification was carried out in a reaction mixture containing 5-μl iQ SYBR Green Supermix (Bio-Rad), 1 μl of primers (mix of forward and reverse primer, 500 nM), and 4-μl cDNA (diluted 20-fold) in triplicate for each sample and gene. The PCR program started with polymerase activation at 95°C for 3 min, followed by 39 cycles of cDNA denaturation at 95°C for 10 s, annealing and amplification at 60°C for 30 s, and a melt curve analysis at 65–95°C with an increment of 0.5°C and 5 s per step at the end.

The relative gene expression and FC were determined using the threshold cycle value (C_t_) and efficiency-based 2^−ΔΔC^_T_ method described by Ganger *et al.* [116], with a modification where log_2_ transformation was applied instead of log_10_. The reaction efficiencies were calculated using real-time PCR miner [117] averaged for each gene for tissue and treatment. Actin and eIF4A were employed as internal reference genes for normalization. The log2 FCs (log_2_FC) were visualized in a heatmap, depicting the upregulation and downregulation of the analyzed genes. Data processing and visualization were performed in R v4.2.2, utilizing the RColorBrewer v1.1–3, dplyr v1.1.4, and EnrichedHeatmap v1.28.1 packages.

### Pore-C library preparation and nanopore sequencing

RE-Pore-C libraries were prepared according to the ONT Plant Pore-C protocol using the restriction enzyme DpnII. The enzyme was heat denatured after overnight incubation and cross-linked DNA clusters were ligated in proximity. The protein was then degraded to remove cross-links, releasing the chimeric Pore-C dsDNA polymers. DNA concentration and purity were determined as described above. Samples with a low A_260/230_ ratio (indicating the presence of polysaccharides) were cleaned up by 1:1 phenol/chloroform extraction and ethanol precipitation. Genomic DNA fragments were damage repaired, end repaired, and A-tailed using NEBNext FFPE DNA Repair Mix and the NEBNext Ultra II End Repair/A-Tailing Module. Libraries were constructed using the ONT Ligation Sequencing Kit (SQK-LSK114) and were sequenced using two R10.4.1 PromethION flow cells. The runtime was set to 100 h in accurate speed mode (260 bps).

### Genome assembly

After basecalling, any remaining adapters were removed from the genomic DNA using Porechop v0.2.4 [118] and the longest reads (> 50 kbp) were selected using Filtlong v2.9.1 (https://github.com/rrwick/Filtlong) and setting ‘—min_length 50 000’. The filtered ONT reads were assembled with the PacBio single-molecule real-time (SMRT) reads using hifiasm v0.19.6-r595 [22, 119] in hybrid mode where the nanopore data were supplied with setting ‘–ul’ and an estimated genome size of 0.8 Gbp. For *k*-mer analysis, we used Jellyfish v2.2.10 (https://github.com/gmarcais/Jellyfish) and GenomeScope (http://qb.cshl.edu/genomescope/) with default parameters (*k* = 23). PurgeHaplotigs v1.1.2 [120] was used to remove duplicate sequences. The Pore-C sequences were virtually digested using pore-c-py ‘digest’ (https://github.com/epi2me-labs/pore-c-py) and aligned with the previously generated genome using minimap2 v2.25 [121]. Aligned files were annotated using pore-c-py ‘annotate’ in paired-end mode (with settings ‘–monomers–chromunity–chromunity_merge_distance-1–paired_end–filter_pairs–paired_end_minimum_distance 100–paired_end_maximum_distance 200’) and duplicates were removed using picard v2.21.8 with setting ‘MarkDuplicates’ (https://github.com/broadinstitute/picard) and default parameters. The aligned Pore-C data were then used by yahs scaffolder v1.1 [122] to scaffold the contig assembly, followed by manual curation using Juicebox Assembly Tools v1.11.08 [123]. Quality was assessed using Quast v4.6.3 [124] and BUSCO v5 [26] to provide information on assembly completeness and genome quality.

Finally, the two resulting haplotypes constructed by hifiasm were scaffolded to the haploid genome representation using RagTag v2.1.0 [125] using correct and scaffold with default parameters to obtain complete chromosome scale haplotypes. Synteny and structural rearrangements were visualized using Syri v1.7.0 [126] with default parameters.

SNPs were called using Freebayes v1.3.6 [127] with DNA short reads and default parameters for diploid organisms. SNPs were then annotated using SNPEff v5.2c [128]. Furthermore, SNPs were functionally annotated using MapMan protein classes, and an enrichment analysis based on these classes was conducted using R Studio (version 2023.06.0, [129]).

### Genome analysis

Methylated site probabilities from mapped PacBio reads were generated using pb-CpG-tools v2.3.2 (https://github.com/PacificBiosciences/pb-CpG-tools) with a threshold of 75% for methylated motifs and were plotted with gene density, GC content and TE abundance, heterozygous SNP rate, stop gained, and stop lost SNP Codons in 1-Mb windows using the R circlize package v0.4.15 (Gu *et al*., 2014). Furthermore, we used the EDTA [24] pipeline and LTR_retriever ‘LAI’ v2.9.8 [130], CRAQ v1.0.9α [25], and Meryl v1.4 followed by Merqury v1.3 [23] for quality validation using the Pacbio reads. We used blastn in BLAST v2.15.0+ [131] to compute the synteny of 73 SNPs common to the SCRI 9328 and MP7 linkage map [2] and our genome assembly. Quality parameters were visualized using R Studio base v2023.06.0 [129]. The scaffolded genome assembly was annotated using Helixer v0.3.1 [132] with the model ‘land_plant_v0.3_a_0080.h5’ and StringTie v2.2.1 [133] with RNA-Seq Illumina short-reads and cDNA nanopore RNA-Seq reads. Splice junctions were detected with Portcullis v1.2.0 (https://github.com/EI-CoreBioinformatics/portcullis) and analyzed with the Helixer and StringTie annotation results in Mikado v2.3.0 [134]. The transcriptome and proteome were extracted from the annotated genome using Gffread v0.12.4 [135]. The resulting protein sequences were used in Mercator and MapMan4 v5.0 [27] for functional gene annotation. Transcriptome quality was assessed using BUSCO v5.

### Evaluation of the drought stress experiment

The cDNA long reads were mapped onto the transcriptome assembly using Minimap2 assembler v2.25 (settings ‘-ax splice -ts’) [121]. The read count was quantified using Salmon v1.10.1 [136]. Paired-end Illumina short reads for leaf samples of young plants were pseudo-mapped and quantified using Salmon v1.10.1. DEGs were identified using edgeR v3.40.1 [137], and we calculated the *P* values and adjusted *P* values (FDR). The counts per million (CPM) data were evaluated by principal component analysis (PCA). Furthermore, overrepresentation analysis based on MapMan annotation was used to find drought stress-related pathways in leaves and roots, and candidate genes were then selected.

### Metabolite analysis

Metabolites in leaf and root samples of adult plants and leaf samples of young plants were analyzed by gas chromatography-masspectrometry (GC–MS) [138, 139]. Mass spectra were compared with the Golm Metabolome database for identification [140]. Metabolomic data were log_2_ transformed, and the average values were calculated for control and treated tissues. The data were normalized against the average values of the control samples. To assess the significance of differences, we calculated the log_2_ FC (log_2_FC), *P* values (*t*-test), and adjusted *P* values (FDR, Benjamini-Hochberg). The statistical tests were used to detect significant differences between the control and stressed samples for each metabolite. The data were visualized using enhanced volcano plots.

### Data integration

A database was built using MariaDB v10.6.5 to establish a connection between the transcriptome and metabolome. Protein sequences representing land plant genes with known functions were extracted from the UniProt SWISSPROT database (May 2022) and linked to MapMan protein classes. These annotated genes were supplemented with associated enzymatic reactions from the RHEA database (https://www.rhea-db.org/). The substrates and products of each reaction were linked to their CHEBI (https://www.ebi.ac.uk/chebi/) and PubChem (https://pubchem.ncbi.nlm.nih.gov/) IDs. We then used the hierarchical organization of MapMan bins, which categorize major biological processes as top-level bins and their subprocesses as child bins, to establish connections between the enzymatic reactions and their associated metabolites. This was achieved by identifying closely related DEGs that associated with these processes and their corresponding bins. By linking the DEGs and their MapMan protein functions to the corresponding metabolites, we established a comprehensive understanding of the relationship between gene expression, biological processes, and metabolites. Genes associated with metabolites were filtered by FDR < 0.05 and pathway-related genes were selected. Statistical analysis was conducted using R Studio base v2023.06.0 [129].

## Supplementary Material

Web_Material_uhae313

## Data Availability

Processed data are available in the supplementary data including read data summaries (S12) and raw sequencing data, and the genome will be available on EMBL-EBI (PRJEB77865). In addition, we added in the supplementary data the genomes and their annotation to the PLANTdataHUB [141] https://git.nfdi4plants.org/usadellab/ribes_nigrum_genome to make them accessible before publication.
