# Supplementary figures and images for: A full genome assembly reveals drought stress effects on gene expression and metabolite profiles in blackcurrant (Ribes nigrum L.)
Source: Hortic Res. 2024 Nov 11;12(2):uhae313. doi: 10.1093/hr/uhae313 (PMC11817994; doi:10.1093/hr/uhae313)

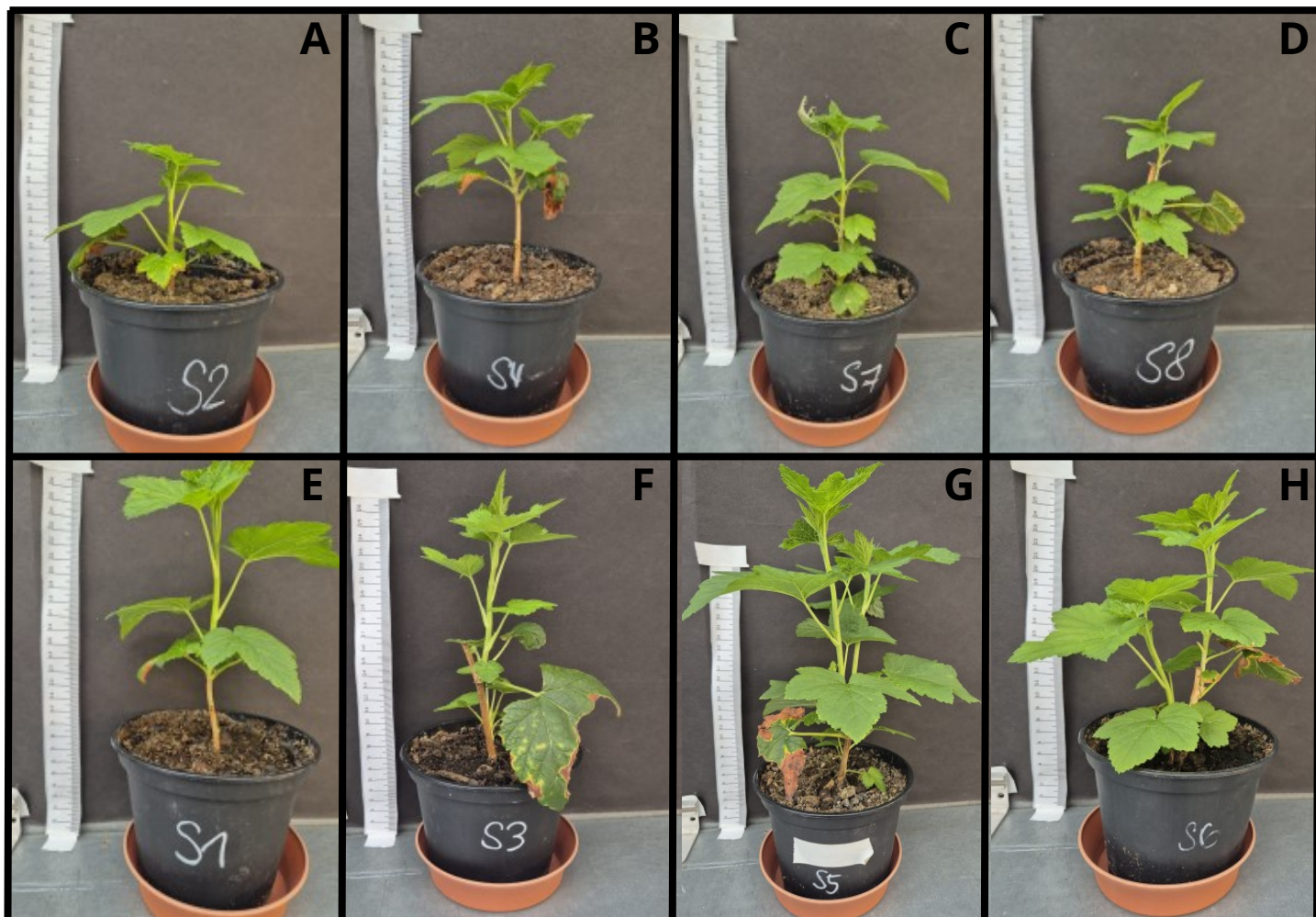

Supplement: Web_Material_uhae313 [file web_material_uhae313.zip › Supplemental_Figure5.pdf]
